# Mekong Basin Disease Surveillance (MBDS): A Trust-Based Network

**DOI:** 10.3402/ehtj.v6i0.19944

**Published:** 2013-01-25

**Authors:** Bounlay Phommasack, Chuleeporn Jiraphongsa, Moe Ko Oo, Katherine C. Bond, Natalie Phaholyothin, Rapeepong Suphanchaimat, Kumnuan Ungchusak, Sarah B. Macfarlane

**Affiliations:** 1 Department of Disease Control, Ministry of Health, Lao PDR; 2 Department of Disease Control, Ministry of Public Health, Thailand; 3 MBDS Foundation, Thailand; 4 The Rockefeller Foundation's Asia Office, United States; 5 The Rockefeller Foundation's Asia Office, United States; 6 International Health Policy Programme, Ministry of Public Health, Thailand; 7 Department of Epidemiology and Biostatistics, School of Medicine, University of California San Francisco, United States

**Keywords:** MBDS, trust-based collaboration, Mekong Basin, infectious disease surveillance, regional network, cross-border, human resource, outbreak investigation and response, FETP, epidemiological capacity

## Abstract

The Mekong Basin Disease Surveillance (MBDS) network was formally established in 2001 through a Memorandum of Understanding signed by six Ministers of Health of the countries in the Greater Mekong sub-region: Cambodia, China (Yunnan and Guangxi), Lao PDR, Myanmar, Thailand and Vietnam. The main areas of focus of the network are to: i) improve cross-border infectious disease outbreak investigation and response by sharing surveillance data and best practices in disease recognition and reporting, and by jointly responding to outbreaks; ii) develop expertise in epidemiological surveillance across the countries; and iii) enhance communication between the countries. Comprised of senior health officials, epidemiologists, health practitioners, and other professionals, the MBDS has grown and matured over the years into an entity based on mutual trust that can be sustained into the future. Other regions have started emulating the network's pioneering work. In this paper, we describe the development of MBDS, the way in which it operates today, and some of its achievements. We present key challenges the network has faced and lessons its members have learned about how to develop sufficient trust for health and other professionals to alert each other to disease threats across national borders and thereby more effectively combat these threats.

## Introduction

**Fig. 1:**
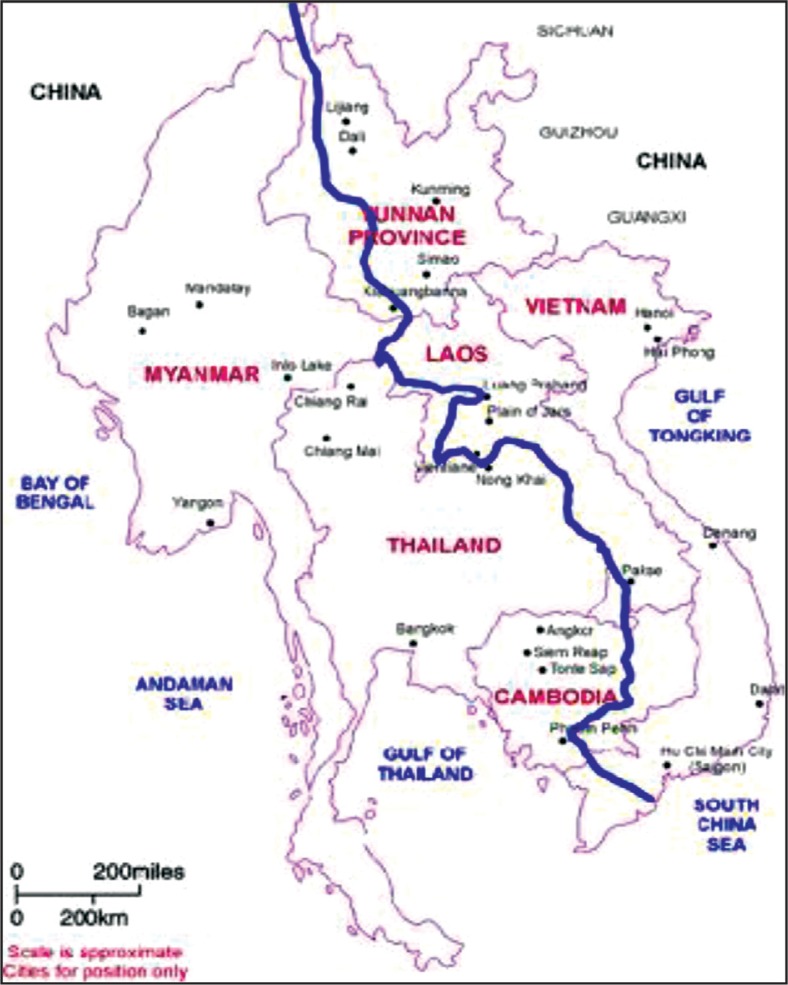
Greater Mekong Sub-region. Source: United Nations Environment Programme (UNEP) (1).

In February 1999, representatives of the six bordering countries through which the Mekong river runs – Cambodia, China (Yunnan and Guangxi), Lao PDR, Myanmar, Thailand, and Vietnam (Figure 1) – convened in Bangkok, Thailand, and agreed to work closely to combat disease outbreaks in the region (sometimes referred to as the Greater Mekong Sub-Region). At this meeting, facilitated by the Rockefeller Foundation (RF) (2), participating epidemiologists and policy makers proposed creation of the Mekong Basin Disease Surveillance (MBDS) network and, upon returning to their respective countries, obtained approval from their ministers of health to establish MBDS. Development of MBDS was in direct response to the 1997 memorandum of understanding between the World Health Organization (WHO) and the Association of South East Asian Nations (ASEAN) which identified disease prevention and control as a priority for inter-country collaboration; and it coincided with a “wake-up call” from the WHO Director-General “to the world's governments, decision makers, and the private sector to take action against infectious disease before it is too late” (3).

The flow of the Mekong river and its tributaries provide environmental continuity and shared livelihoods, but also common health challenges for people of diverse nationalities closely linked by cultural, historical, and linguistic ties. For example, cholera is a constant threat to livelihoods in all countries in the region; its reporting is politically sensitive particularly because of its threat to tourism (4). In 1999, when MBDS was coalescing into a network, there was a serious outbreak of cholera in a remote northern province of Cambodia bordering Vietnam during which 874 cases and 56 deaths were reported (5). Cambodia recognized that not only did it need to strengthen community-based surveillance, but also that it could better contain such epidemics if Cambodian and Vietnamese epidemiologists and officials worked together.

Health status in the region also reflects national as well as regional economic and political diversity. So while the spectrum of communicable diseases in the six countries is qualitatively similar, incidence varies considerably. For example, in 2010 the incidence of tuberculosis in China, Lao PDR and Thailand ranged from 78 to 137 cases per 100,000 population, which was about half the incidence in Cambodia, Myanmar and Vietnam where it ranged from 199 to 347 per 100,000 (6). In 2010, infant mortality rates ranged from 42 to 50 per 1000 live births in Cambodia, Lao PDR and Myanmar, compared to 11 to 19 per 1,000 live births in Thailand, China and Vietnam.

The context in which MBDS emerged differed from the one in which it operates today. People living in the six countries were familiar with the dangers of communicable diseases – such as multi-drug-resistant malaria, dengue hemorrhagic fever, sexually transmitted diseases, HIV/AIDS, tuberculosis, Japanese encephalitis, visceral leishmaniasis, hepatitis E and cholera. Also, while there was a strong tradition of public health and epidemiological intelligence in the region, particularly in Thailand, the lower income countries were still developing human resources to strengthen their health systems. National systems for controlling outbreaks of infectious diseases were weak and understaffed. Moreover, although international aid supported vertical reporting to WHO of national data for specific diseases such as malaria, tuberculosis and HIV/AIDS, epidemiologists found it difficult to communicate politically and economically sensitive information horizontally between countries or via the internet. The six countries set up MBDS with three main areas of focus: i) to improve cross-border infectious disease outbreak investigation and response by sharing surveillance data and best practices in disease recognition and reporting and by jointly responding to outbreaks; ii) to develop expertise in epidemiological surveillance across the countries; and iii) to enhance communication between the countries. Today, MBDS plays a key role in disease control in the region, enhancing efforts by governments, WHO, and U.S. Centers for Disease Control and Prevention (CDC) to build national and regional capacity to face the dangers of new disease outbreaks such as SARS and avian influenza H5N1 (7).

## Governance and Values

**Fig. 2:**
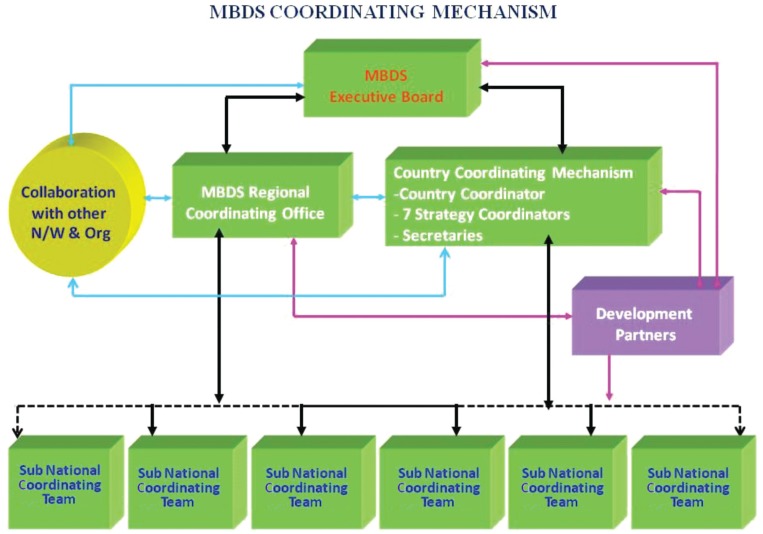
MBDS coordinating mechanism (N/W & Org=networks and organizations). Source: MBDS.

The health ministers of each MBDS member country signed two memoranda of understanding, the first in 2001 and the second in 2007, to provide an agreed framework for the governing structure and processes of the consortium (Figure 2): each country would be represented by a country coordinator; the country coordinator would work closely with cross-border coordinators responsible for designated sites where the extent of cross-border movement could lead to disease outbreaks; a network secretariat would organize regular meetings of country and cross-border coordinators and support all members in the network's activities; and an MBDS Executive Board, made up of one policy maker at the senior level from each member country, would set policy and link the network to higher levels of government. Country coordinators are usually epidemiologists based in the health ministry departments responsible for disease surveillance; the MBDS Secretariat is hosted by the Thai Ministry of Public Health, which provides office space and other support.

The leaders of MBDS realized the importance of institutionalization of the network and have been working towards this since 2008. After a great deal of discussion and brainstorming, the network decided to turn itself into a legal entity. In January 2012, MBDS formally registered in Thailand as a foundation. The main purpose of this new arrangement is to mobilize funding so that MBDS can continue its activities unhindered. MBDS formed a new board with representatives of the six countries and a few “invited” members, and is recruiting a new director with relevant experience to help the MBDS Secretariat.

While MBDS operates within an agreed governance structure and according to agreed processes, it is driven by informal trust-based relationships between MBDS member countries. Although mutual trust is the core value of the network, this trust did not appear overnight, but grew steadily. Joint activities have gradually built a platform for regular interactions among the country coordinators, local cross-border teams, and other stakeholders to learn about each other as professionals and as individuals and to foster a sense of community. A decade after its birth, all founding MBDS leaders are still actively involved in network activities. This crucial continuity of leadership is also apparent at the border sites, for example, the Mukdahan and Savanhnaket health staff on the Thai-Lao borders regularly communicate with each other informally, as villagers and patients frequently cross the border. In a trip to the Bokeo and Chiang Rai site, a colleague working for one of MBDS's international partners (8) observed the cordial relationship between the staff of the local health departments of Lao PDR and Thailand and the active exchange of information taking place between them using modern technologies. Language is often a barrier in communicating, but this was not the case as the two countries understood each other's languages.

The informal trust-based relationships between MBDS member countries complement the formal vertical MOU-based relationship and WHO/International Health Regulations reporting structures (9, 10) - especially important as the MBDS countries cross two WHO regions (i.e., the South-East Asia Region and Western Pacific Region). Thus, the governing structure of the MBDS is – like a piece of “social fabric” that is skillfully woven by crisscrossing horizontal (informal trust-based relationships) and vertical (formal and official relationships) threads.

## International Organizations Partnering With MBDS

A number of partners contributed to the development of MBDS. The RF was the primary and first donor and provided core support from 1998 to 2012. Other major donors and partners have included: the Agence Française de Développement (AFD); Asia-Pacific Economic Cooperation Emerging Infections Network (APEC EINet) maintained by the University of Washington; ASEAN Plus Three Emerging Infectious Disease (EID) Programme under the auspices of the Association of Southeast Asian Nations (ASEAN) Secretariat and funded by the Australian Agency for International Development (AusAID); Asian Development Bank Greater Mekong Sub-regional Communicable Diseases Control Project (ADB-GMS-CDC); Innovative Support to Emergencies, Diseases and Disaster (InSTEDD); Kenan Institute Asia; Nuclear Threat Initiative Global Health and Security Initiative (NTI GHSI); Program for Monitoring Emerging Diseases (ProMED), an activity of the International Society for Infectious Diseases (ISID); RAND Corporation; World Health Organization (WHO); the World Organization for Animal Health (OIE); and the United Nations System Influenza Coordination (UNSIC).

MBDS has itself contributed to the development of other similar regional networks, through the active involvement of its members. The spontaneity of the relationships between MBDS members was instrumental in forming the ASEAN Plus Three Centre for Emerging Infectious Disease where 6 of the 13 members are MBDS members (11), the ASEAN Plus Three Field Epidemiology Training Network (12), and the Asian Pacific Emerging Infectious Diseases Research Network (APEIR) with 3 of its 5 members from MBDS countries (13). These networks were also linked by MBDS's to its participation in Connecting Organizations for Regional Disease Surveillance (CORDS).

## Strategies and Achievements

Over time, MBDS priorities have evolved to reflect its three phases of development (Table 1) (see reference 17 for a discussion of the three phases). During the first phase (1999 to 2003), country representatives met regularly, set up committee structures, established the MBDS Coordinating Office in Thailand, and began developing capacity.

**Table 1:** Timeline of significant epidemiological events and regional collaborative response during the initial 12 years of MBDS.

Year	Outbreaks/epidemiological events	Regional collaboration
**MBDS Phase 1: Formation and development of MBDS, building trust and capacity**		
1999	Cholera outbreak in Vietnam	International Field Epidemiology Training Program (IFETP)-Thailand responded (14)
2000	The first licensed rotavirus vaccine was withdrawn in 1999	Asian Rotavirus Surveillance Network was initiated in 2000 (15)
2002	Severe Acute Respiratory Syndrome (SARS) outbreak in China	Information exchange within the sub-region on cases and travelers
2003	SARS and H5N1 avian influenza outbreaks in China, Vietnam, and Thailand	Initiation of ASEAN Plus Three work plan
**MBDS Phase 2: Building capacity, setting up cross-border sites and preparing for pandemics**		
2004	Avian influenza outbreak in Prachinburi, Thailand; Tsunami in Thailand	Human and animal sectors started working together in all countries; ASEAN Plus Three Ministers of Health joint declaration on protection and control avian influenza (16); surrounding countries assisted Thailand cope with the tsunami.
2005	Avian influenza and botulism outbreaks in Thailand; human plague outbreak in China	IFETP-Thailand recruited a veterinarian.
2006		In-country regional simulation exercises to prepare for pandemics
2007		Regional pandemic preparedness simulation exercise
**MBDS Phase 3: Seven core strategies based on needs identified during phases 1 and 2**		
2007	Cholera outbreak in Thailand; Chigunkunya outbreak and melamine contamination of milk products in China	Joint cholera outbreak investigation between Thai and Lao teams (Text Box 2)
2008	Cyclone *Nargis* in Myanmar	ASEAN/MBDS assistance to Myanmar
2009	Pandemic H1N1 in all MBDS countries and measles in Hanoi, Vietnam	Communication on outbreak detection and responses; and FAO started an International Field Epidemiology Training Program for Veterinarian (FETPV) in this region.
2010	Hand foot and mouth disease in China and dengue hemorrhagic fever in Lao PDR	Start of ASEAN Plus three Field Epidemiology Training Network (11)
2011	Tsunami in Japan; flooding in Thailand	Tele-conferences between ASEAN Plus Three countries on hand foot and mouth disease and to respond to the flooding

The arrival of avian influenza H5N1 in late 2003 and the growing threat of an imminent influenza pandemic signaled the need for new methods to strengthen preparedness nationally and regionally. Thus, during the second phase (2004 to 2007), while MBDS representatives continued to build capacity and worked together to set up multiple cross-border projects, they undertook regional simulation exercises to plan for pandemics. Specifically, as described in Text Box 1, MBDS and its partners (RF, NTI, CDC and RAND) designed and carried out a series of “tabletop” simulation exercises to: i) explore national and regional cross-border strategies in pandemic emergencies; ii) identify priorities to improve preparedness and response; and iii) develop recommendations to help guide further MBDS programming and donor investments. These exercises informed development of the MBDS Action Plan (2008–2013) described in Table 2, (21, 22).

**Table 2:** MBDS Core Strategies

Strategy	Country responsible	Major activities and achievements	Partner support (See text for full names)
1.Enhance cross border communication and information exchange	Lao PDR	Set up 16 cross-border sites for disease control cooperation and disease surveillance information exchange.	ADB-GMB-CDC, ASEAN Plus Three EID Programme, K.L. Asia, RF
2. Improve the human-animal sector interface and strengthen community surveillance	Vietnam	Shared experiences of collaboration between animal and human sectors working between the Cambodia and Lao border provinces.	ADB-GMB-CDC, ASEAN Plus Three EID Programme, K.L. Asia, ProMed, RF, WHO
3. Develop human resources and strengthen epidemiological capacity	Thailand	Conducted joint investigations of dengue hemorrhagic fever and H5N1 influenza cases 2007; trained 41 FETP trainees and 6 FETP trainers; agreed on human resource development indicators (18); and established FETP programs in Vietnam (2009), Lao PDR (2009), and Cambodia (2011).	ADB-GMB-CDC, APEC EINet, ASEAN Plus Three EID Programme, INSTEDD, K.L. Asia, NTI GHSI, ProMed, RAND, RF, University of Washington Center for Excellence in Public Health Informatics, WHO
4. Strengthen capacities for information and communications technologies	Cambodia	Established GeoChat, a SMS-based real time surveillance reporting system across the MBDS countries, in Mukdahan, Thailand.	ADB-GMB-CDC, APEC EINet, InSTEDD, K.L. Asia, ProMed, RF, University of Washington Center for Excellence in Public Health Informatics,
5. Strengthen laboratory capacity	China	Assessed capacity and needs of 40 laboratories in six MBDS member countries in Cambodia, Guangxi, Yunnan, Lao PDR, Myanmar, Thailand, and Vietnam.	ADB-GMB-CDC, AFD, ASEAN Plus Three EID Programme, InSTEDD, K.L. Asia, NTI GHSI, RF, WHO
6. Strengthen risk communications	Myanmar	Documented experience of national level disaster management collaboration with ASEAN and UNICEF	ADB-GMB-CDC, ASEAN Plus Three EID Programme, ProMed, RF, WHO,
7. Conduct and apply policy research	Collective	Assessed pandemic influenza response among MBDS countries (19); and the potential of regional infectious disease surveillance networks to facilitate implementation of international health regulations (20).	ADB-GMB-CDC, ASEAN Plus Three EID Programme InSTEDD, RAND, RF

***Text Box 1.* MBDS regional tabletop simulation exercises**

In 2006, after the outbreak of H5N1 avian influenza in the region, MBDS countries recognized the urgent need to strengthen national and regional preparedness to face new pandemic threats. From August to October 2006, MBDS brought together representatives from the public health, agriculture, foreign affairs, defense, and finance sectors and from WHO, OIE and UNSIC to develop scenarios and plan and carry out a series of tabletop simulations within each country. In March 2007, Cambodia hosted the first ever regional level pandemic preparedness simulation exercise, attended by 85 participants. The simulation focused on pandemic capabilities with the greatest relevance to transnational cooperation: surveillance and information sharing, disease prevention and control, and communication. These exercises contributed to greater confidence and ownership at the national level; and improved communication, trust and collaboration at the regional level. They also led to other applications of the tabletop simulation methodology. Myanmar used the methodology in 2008 to plan its response to a severe outbreak of diarrhea; Lao PDR used it in 2009 to plan medical emergency preparedness while hosting the Southeast Asian Games; and Vietnam conducted tabletop simulation exercises in 2009 and 2010 for the control of animal-to-human disease transmission.

During the third phase (2008 to 2011), network activities fell within seven core strategies, each strategy led by one country based on its capacity or its interest to develop the relevant capacity: (i) enhance cross-border communication and information exchange; (ii) improve the human-animal sector interface and strengthen community surveillance; (iii) develop human resources and strengthen epidemiological capacity; (iv) strengthen capacities for information and communications technologies; (v) strengthen laboratory capacity; (vi) strengthen risk communications; and (vii) conduct and apply policy research. (See Table 2 for a summary). Here, we describe two of these core strategies in detail (i and iii).

### Enhancing Cross-Border Communication

**Fig. 3:**
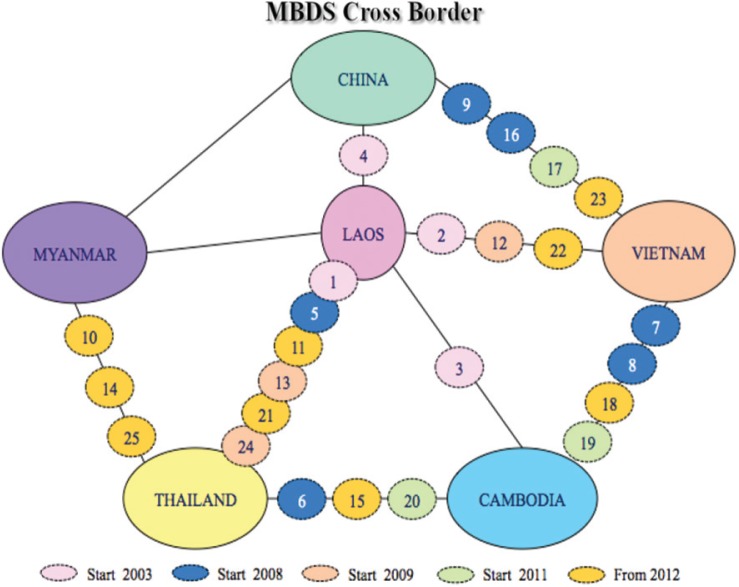
Location of cross-border sites developed from 2003 to 2012 (“From 2012” sites include some sites still pending). Source: MBDS.

MBDS established 16 functioning cross-border sites at major crossings between the six countries (Figure 3). The purpose of these sites is to facilitate cross-border teams of health, customs, immigration, and border officials to undertake joint outbreak investigation and response. National MBDS coordinators and adjacent provincial site coordinators exchange routine surveillance data about suspected outbreaks: daily for any case of influenza H1N1, acute flaccid paralysis (AFP, i.e., potential polio), SARS, cholera/severe diarrhea, encephalitis, tetanus, meningitis, diphtheria, and public health emergencies of international concern (PHEIC); weekly for cases of leptospirosis, chikungunya, dengue fever, typhoid fever and measles; monthly for cases of malaria and pneumonia; and less frequently for cases of HIV/AIDS and tuberculosis. Field Epidemiology Training Program (FETP)-Thailand works in collaboration with the rapid response teams at cross-border areas to evaluate and strengthen their joint surveillance and response activities (23, 24). These teams investigated and contained dengue fever outbreaks between Lao PDR and Thai provincial sites in January 2005 (Khanthaboury Province) and June 2006 (Xaythuthong province); a typhoid and malaria outbreak between provincial sites in Lao PDR and Vietnam (Savannakhet and Quang Tri provinces) in 2006; and an avian flu incident in Lao PDR after detecting an infected Laotian in Thailand in 2007 (Text Box 2). In May 2008, the Thai and Myanmar MBDS teams worked together to combat the effects of Cyclone Nargis when it hit Myanmar (Text Box 3).

***Text Box 2.* Joint outbreak investigation of a human H5N1 influenza case by Rapid Response Team (RRT) in Lao PDR, and Surveillance and Rapid Response Team (SRRT) in Thailand, 2007**

Following an announcement of an avian influenza H5N1 outbreak among poultry in Nong Khai Province in Thailand, which borders Lao PDR, a similar outbreak among poultry was confirmed in Vientiane, Lao PDR. The Lao PDR investigation team had also identified three suspected human cases, one reportedly admitted to Sethathirath Hospital, Vientiane. The Lao and Thai teams worked closely with each other and with the Lao PDR and Thai Ministries of Health to facilitate confirmation of the first human avian influenza case in Lao PDR. After being admitted to the hospital in Vientiane on February 15, the index case was transferred to Nong Khai on February 17. The Lao RRT notified the Nong Khai Provincial Health Office of the transfer on February 19. The Lao RRT and Thai SRRT initiated a joint Lao-Thai investigation on February 20, with specimens for H5N1 testing collected that day and tested the next day at the Thai NIH. They tested positive. The Lao PDR and Thai Ministries of Health participated in the joint investigation on February 24–25, and the Lao PDR Ministry of Health publicly announced the first human case of infection with H5N1 virus on February 27. This collaborative effort strengthened the surveillance system, public health workforce, and border practices on both sides.

***Text Box 3.* MBDS response to Cyclone Nargis**

**Fig. 4:**
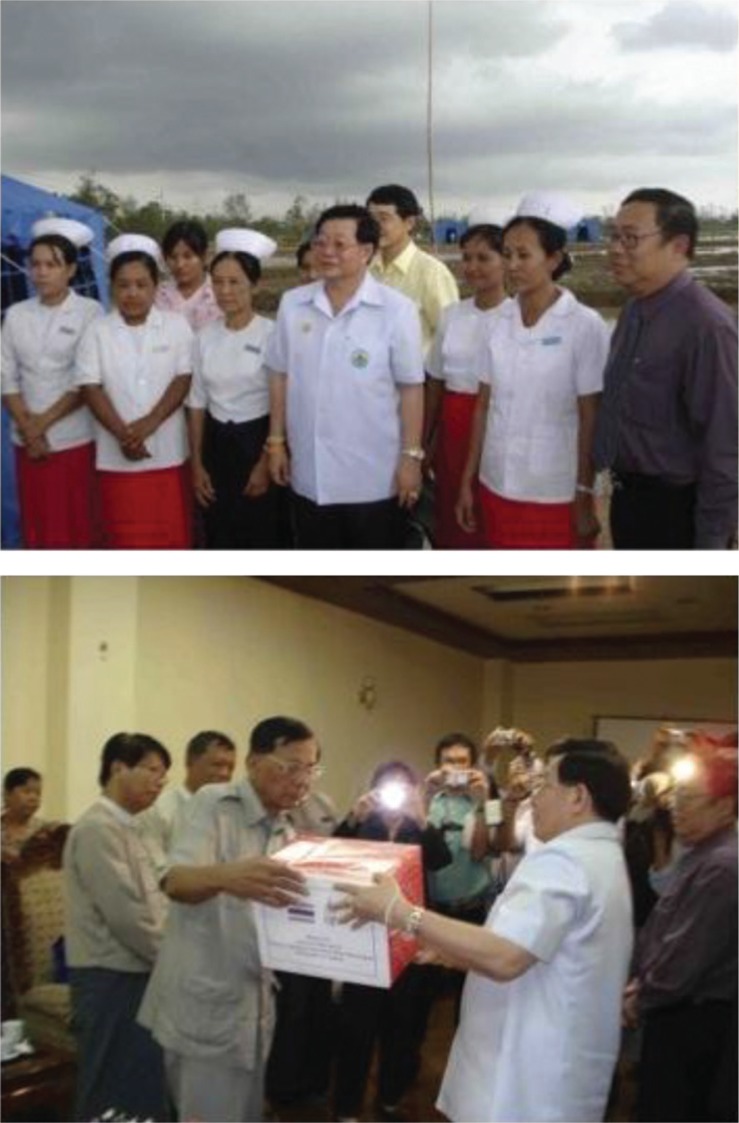
Images of MBDS post-disaster relief aid to Cyclone *Nargis*-affected area in Myanmar. Source: MBDS.

On May 2, 2008, Cyclone *Nargis* struck the Irrawaddy Delta of Myanmar, causing the worst natural disaster in recorded history and resulting in at least 138,000 fatalities and destruction of property estimated at over U.S.$10 billion. Lack of relief facilities led authorities to fear a “second wave” of fatalities from diseases (25). A rapid response team of physicians, psychologists, and environmentalists from MBDS Thailand and Thai Red Cross assisted victims of the cyclone in the Myuangmya region, approximately 46 miles from the hardest hit area (Figure 4). The team was concerned about outbreaks of infectious respiratory diseases that might spread to neighboring countries without immediate intervention. No outbreaks were detected, except a few cases of respiratory illnesses. This post-disaster relief effort may not have been mobilized or succeeded in its mission without the existing relationships and collaborative procedures formed through the trust-based MBDS network.

### Strengthening Epidemiological Capacity

MBDS and its partners have organized extensive training for member countries under the leadership of the International Field Epidemiology Training Program (IFETP)-Thailand. IFETP-Thailand, the U.S. CDC and WHO provided two-year FETP training to mid-career public health professionals from all six member countries (plus Malaysia and Bhutan) and on-the-job training to FETP alumni to become FETP trainers. Additionally, with support and commitment from the U.S. CDC, the WHO Western Pacific Regional Office, and other development partners, a competency-based epidemiology training program, similar to FETP, was launched and grew promisingly in Vietnam, Lao PDR and Cambodia, with graduates from IFETP-Thailand serving as trainers for these national programs. Other training programs that MBDS has been involved with include postgraduate training for physicians from Lao PDR and China, with the support of NTI and the Prince of Songkhla University, Thailand; short-course training on laboratory management, geographical information systems, and use of epidemiological software for members of the cross-border rapid response teams, with support from the RF; short-course training courses in surveillance and response organized by the ministries of health in Cambodia, Lao PDR, Vietnam, and China; and a variety of training projects supported by RF and NTI through MBDS.

Also to enhance epidemiological capacity, in 2009 IFETP-Thailand reviewed each member country's human resources capacity in epidemiology. Based on the review, Thailand developed and published a set of 20 indicators for human resource development in epidemiology (18) and conducted a follow-up review in 2012.

A number of evaluations of the MBDS have been carried out. The most recent was conducted by an independent team led by SEAMEO-Tropmed. It confirmed the relevance and efficiency of the MBDS network as it has responded to the needs of the countries in the region in terms of disease surveillance, capacity building, and outbreak investigation and containment. This review also highlighted the various impacts the network has had, including the creation of trust between countries (26).

## Lessons Learned

Here we highlight two key lessons learned over the 13-year history of MBDS. First, there is a big difference between running a project and running a longer term trust-based collaboration. A project has an end date and responsible persons whose aim is to achieve the agreed results in due time no matter what will happen. A collaboration whose goal is to build trust requires more time and does not inherently have an end date. Trust cannot be established without the type of common understanding among member countries that can only be gained through continuous engagement. Only by working with each other over time, for example by making decisions about difficult situations through “consensus” and by rotating leadership of the network on an annual basis, did MBDS establish trust and derive strength from it. The mutual trust established over these years is a strong platform for sustaining MBDS collaboration into the future (27).

A second key lesson learned is the value of working with official structures. Most disease surveillance systems in the region and elsewhere are mainly run by government systems. It was a prudent decision at the beginning to place MBDS within the official governance structures of each country. For example, the fact that country coordinators are government officials who already know each other and are friendly to each other facilitates MBDS operations and makes MBDS contributions integral to government operations.

## Moving Forward

Because building trust takes time, continuity of leaders and sustained support from development partners is crucial. Long-term commitment from the RF and other partners has significantly contributed to the network's success. The future will depend on how the health leaders of the six countries evaluate the continued relevance of the network in constantly changing contexts and how the network's new legal entity, the MBDS Foundation, carves out its role. It is expected that the newly formed MBDS Foundation, in addition to solidifying and institutionalizing cooperation, will serve as the network's financial arm by mobilizing resources from different funding agencies, including from governments of member countries. Also contributing to network sustainability are the large number of development agencies which have used the MBDS mechanism and structure for their own disease surveillance network; the ministries and provincial health offices in bordering countries or provinces which have the network's culture of cross-border information exchange; and the large number of FETP graduates now occupying important positions in member country ministries of health.

Since its inception, MBDS has been tested by historic health events. After several years of interactive learning through joint actions, individual leaders and their staff have firmly established a mutual trust and overcome many difficult challenges. A good example is the joint outbreak investigation into an H5N1 case between Lao PDR and Thailand which was implemented without even a formal document or official agreement (Text Box 2). In the first three years of MBDS existence, when mutual trust was not strong, sharing of outbreak information was difficult. However, as mutual trust improved, cross-border data sharing dramatically increased. Today, MBDS serves as an exemplary model for regional disease surveillance in other parts of the world, including Southern Africa, Eastern Africa, the Middle East and South Asia.

## References

[1] United Nations Environmental Programme [UNEP] Strategic Environmental Framework for Greater Mekong Subregion. http://www.rrcap.unep.org/lc/cd/html/projects/proj2.html.

[2] The National Bureau of Asian Research Mekong Basin Disease Surveillance (MBDS) Network. http://www.pacifichealthsummit.org/downloads/HITCaseStudies/Functional/MBDS.pdf.

[3] World Health Organization [WHO] (1999). WHO Issues “Wake-Up Call” Against Microbial Threats. Press Release. http://www.who.int/inf-pr-1999/en/pr99-33.html.

[4] Tauxe RV, Branchman ES, Branchman PS, S. A (1998). Cholera.

[5] World Health Organization [WHO] (1999). Cholera in Cambodia. Global Alert and Response (GAR). http://www.who.int/csr/don/1999_05_21/en/index.html.

[6] Health Protection Agency [HPA] World Health Organization (WHO) estimates of tuberculosis incidence by country, 2010. http://www.hpa.org.uk/web/HPAweb&HPAwebStandard/HPAweb_C/1195733837507.

[7] World Health Organization [WHO] Severe Acute Respiratory Syndrome (SARS): Global Alert, Global Response. http://www.who.int/csr/sars/conference/june_2003/materials/presentations/en/sarsglobal170603.pdf.

[8] Rockefeller Foundation (2009). The Mekong Dream Report.

[9] Gresham LS, Pray LA, Wibulpolprasert S, Trayner B (2011). Public–private partnerships in trust-based public health social networking: Connecting Organizations for Regional Disease Surveillance (CORDS). Journal of Commercial Biotechnology..

[10] Gresham L, Ramlawi A, Briski J, Richardson M, Taylor T (2009). Trust across borders: responding to 2009 H1N1 influenza in the Middle East. Biosecurity and Bioterrorism: Biodefense Strategy, Practice, and Science.

[11] Association of Southeast Asia Nations [ASEAN] Information Center on Emerging Infectious Diseases in the ASEAN Plus Three Countries. http://www.aseanplus3-eid.info/.

[12] Association of Southeast Asia Nations [ASEAN] Plus Three Field Epidemiology Training Network. http://www.aseanplus3fetn.net/?s=2&j=background.

[13] Silkavute P, Tung DX, Mallee H, Jongudomsuk (2013). Sustaining a regional emerging infectious disease research network: a trust-based approach. Emerging Health Threats..

[14] International Field Epidemiology Training Program [FETP]-Thailand http://www.interfetpthailand.net/index.php.

[15] Bresee J, Fang ZY, Wang B, Nelson EA, Tam J, Soenarto Y (2004). First report from the Asian Rotavirus Surveillance Network. Emerg Infect Dis.

[16] Bureau of Epidemiology (2004). ASEAN+3 Ministers of Health joint declaration on protection and control avian influenza. Weekly Epidemiological Surveillance Report. 2004 December 3.

[17] Bond KC, Macfarlane S, Burke C, Ungshusak K, Wibulpolprasert S (2013). The evolution and expansion of regional disease surveillance networks and their role in mitigating the threat of infectious diseases outbreaks. Emerging Health Threats.

[18] Jiraphongsa C, Doung-Ngern P, Iamsirithaworn S, Gresham L, Zin T, Moore M (2011). MBDS-FETP and Human Resource Development 2010–2011. http://www.interfetpthailand.net/file/mbds/mbds-fetp_book.pdf.

[19] Moore M, Dausey DJ (2011). Response to the 2009-H1N1 influenza pandemic in the Mekong Basin: surveys of country health leaders. BMC Res Notes.

[20] Kimball AM, Moore M, French HM, Arima Y, Ungchusak K, Wibulpolprasert S (2008). Regional infectious disease surveillance networks and their potential to facilitate the implementation of the international health regulations. Med Clin North Am..

[21] Mekong Basin Disease Surveillance [MBDS] Action Plan (2008–2013). http://www.mbdsoffice.com/regional_plan.php.

[22] Dausey DJ, Moore M (2007). Mekong Basin Disease Surveillance Partners Regional Pandemic Influenza Tabletop Exercise: After Action Review.

[23] Mekong Basin Disease Surveillance [MBDS] Surveillance Evaluation 24 July 09 in Mukdahan and Savannakhet. http://www.svkmuk.com/.

[24] Mekong Basin Disease Surveillance [MBDS] MBDS Cross-Border Sites: Existing and Planned through 2012. http://www.mbdsoffice.com/cb/xb_diagram.pdf.

[25] Myint NW, Kaewkungwal J, Singhasivanon P, Chaisiri K, Panjapiyakul P, Siriwan P Are there any changes in burden and management of communicable diseases in areas affected by Cyclone Nargis? Conflict and Health 5:9. http://www.conflictandhealth.com/content/5/1/9.

[26] Ancheta CA, Mendoza OM, Pachuen O, Perez ES, Richter K, Singhasivanon P (2010). Disease Surveillance Network Initiative Asia.

[27] Moore M, Dausey DJ, Phommasack B, Touch S, Guoping L, Nyein SL (2012). Sustainability of sub-regional disease surveillance networks. Global Health Governance 5(2).

